# Pubic bone osteomyelitis and fistulas after radiation therapy of the pelvic region: patient-reported outcomes and urological management of a rare but serious complication

**DOI:** 10.1007/s00345-024-05155-2

**Published:** 2024-08-01

**Authors:** Margarete Teresa Walach, Anoshirwan Andrej Tavakoli, Greta Thater, Maximilian Christian Kriegmair, Maurice Stephan Michel, Marie-Claire Rassweiler-Seyfried

**Affiliations:** 1 Department of Urology and Urologic Surgery, University Medical Centre Mannheim (UMM), University of Heidelberg, Theodor-Kutzer-Ufer 1-3, 68167 Mannheim, Germany; 2 Department of Radiology and Nuclear Medicine, University Medical Centre Mannheim (UMM), University of Heidelberg, Theodor-Kutzer-Ufer 1-3, 68167 Mannheim, Germany

**Keywords:** Pubic bone osteomyelitis, Urosymphyseal fistula, Pelvic radiation therapy, Cystectomy, Complications

## Abstract

**Purpose:**

This study investigated late urinary adverse events (UAEs) in patients who underwent pelvic radiation therapy, with a focus on occurrence, diagnostic characteristics and the impact of subsequent extirpative surgery with the need of urinary diversion on quality of life.

**Methods:**

A retrospective analysis of 20 patients after pelvic radiotherapy (2016–2022) was conducted. Data included demographics, perioperative details, oncological parameters, and patient-reported outcomes. Imaging (CT, MRI) was examined for early manifestations of late UAEs.

**Results:**

In the study cohort, prostate cancer was the primary malignancy in 85% with a mean radiation dose of 84 Gray over 35 days. Time to diagnosis of late UAEs was 4.0 years post-radiation. Radiological assessment demonstrated a progressive increase in typical CT and MRI features of pubic bone osteomyelitis over time. Surgical interventions, mainly cystectomy, were required with variable outcomes in patient-reported post-surgery quality of life.

**Conclusion:**

Diagnosing and managing late UAEs after pelvic radiation necessitate an understanding of their occurrence, diagnostic features and appropriate management strategies. Early imaging, particularly MRI, is crucial for timely diagnosis and treatment planning. Variable post-surgery quality of life underscores the importance of a multidisciplinary approach in managing late UAEs. The study contributes to understanding these complications and emphasizes their consideration in post-radiation follow-up care.

**Supplementary Information:**

The online version contains supplementary material available at 10.1007/s00345-024-05155-2.

## Introduction

Adjuvant radiation therapy after surgical treatment of urological cancer and primary radiation as curative therapy measures are established therapeutic treatment methods in urological oncology [1–3]. Early detection through improved diagnostic technologies have significantly improved post-diagnosis survival rates. A multidisciplinary approach involving conservative, surgical, and radiation therapies has played a key role [4]. Radiotherapy is estimated to be a highly effective cytotoxic therapy for localized solid malignancies, forming a crucial part of cancer treatment for approximately 50% of all cancer patients [5–7]. One of the disadvantages of radiotherapy is the affection of both cancer and healthy cells in the treated area. As a consequence and due to the anatomy of the pelvis, urological complications occur not only after radiotherapy of urological malignancies but also after radiotherapy of the digestive and reproductive system [8]. Late urinary adverse events (UAEs), such as urosymphyseal fistulas and pubic bone osteomyelitis can occur many years after the initial radiation treatment [9–12]. These burdensome complications present with chronic and unrelenting pubic pain and recurrent urinary tract infections. Although these UAEs are comparatively rare, they can be very serious and significantly degrade quality of life (QoL). Urosymphyseal fistulas and pubic bone osteomyelitis often cannot be treated conservatively, but require major surgical interventions, such as resection of the pubic symphysis, cystectomy and urinary diversion or complicated reconstruction techniques [12–14]. Due to the impaired tissue healing after radiotherapy the surgical treatment of those complications may be a challenge for urologists [12, 15]. Lavien et al. showed improved pain outcomes in patients undergoing surgery for pubic bone osteomyelitis compared to conservative therapy [16].

In general, however, outcomes in these patients, such as health-related quality of life and emotional aspects after definitive surgery are not well described to date.

Despite advancements in radiotherapy reducing radiation damage, late UAEs still occur. Computed tomography (CT) or magnetic resonance imaging (MRI) are necessary diagnostic tools when late UAEs after radiotherapy are clinically suspected [17–19]. However, there is a lack of standardized imaging criteria for an early detection of UAEs [20]. The aim of this study was to assess the natural course of UAEs following pelvic radiation therapy, analyzing radiation dosage, time frames for symptom onset and management of UAEs. Additionally, we aimed to analyze early radiographic manifestations of UAEs and assess QoL following therapeutic interventions involving the urinary tract. By addressing the mentioned aspects, we seek to contribute to a deeper understanding of these complications and hence, to an adequate treatment decision-making.

## Material and methods

### Study population and data collection

Data of 20 patients from a German high-volume tertiary referral university hospital, who underwent pelvic exenteration including cystectomy after radiation therapy of the pelvic region between 2016 and 2022, were retrospectively evaluated. Surgery was only performed for chronic and intractable pubic pain and recurrent urinary tract infections, with the aim of relieving symptoms. None of the patients underwent surgery for tumour recurrence. The collected data included demographic and perioperative parameters, information about the primary malignancy, radiotherapy type, cumulative radiation dose, timing and duration of radiotherapy. Additionally, prospective analysis included health-related quality of life and emotional impact assessed through the EORTC QLQ-C30 and the decision regret scale (DRS) [21, 22]. The EORTC QLQ-C30 assesses functional scales, symptom scales and overall QoL/global health status in a 30-item questionnaire. The scores range from 0 to 100. A high score for functional scales and overall QoL/global health status represents a high level of functioning and QoL, whereas high scores for symptom scales represent high levels of symptomatology. The DRS is a 5-item questionnaire and measures decision regret on a 0–100 scale, where 0 indicates no regret and 100 signifies high regret.

### Radiological assessment

Available sectional radiographic imaging (CT and MRI) was assessed by two uro-radiologists. Three time points of imaging were of interest: (1) after radiotherapy and before onset of symptoms, (2) at first onset of symptoms, (3) preoperatively. One of the radiologists was blinded concerning the date of the imaging. Both radiologists were blinded to the extent of the patient's complaints, but not to the diagnosis itself. The analysis was performed to classify characteristic correlates for pubic bone osteomyelitis, such as pubic bone marrow edema, cleft sign in the pubic symphysis, sclerosis, effusion of the pubic symphysis and pubic bone erosion. Depending on the specific manifestation of those characteristics, points from one (no alteration) to five (very distinct changes) were allotted and a sum score generated. The score ranged from 1 (no signs of pubic bone osteomyelitis) to 5 (very distinct signs of pubic bone osteomyelitis).

### Surgical technique

Extirpative surgery involved the removal of the urinary bladder and the prostate in men. In one case ureteral clipping without cystectomy was performed. Whenever necessary, surgical debridement or resection of the pubic symphysis were performed by an orthopedist. Temporary external fixation was used to stabilize the pelvic ring. In some cases, fistula excision was combined with omentoplasty or protective ileostomy. Omental flaps were attached to fix or fill defects.

### Statistical analysis

All statistical analyses were performed using statistical software JMP^®^ from SAS (version 13 for Windows, SAS Institute Inc.) and GraphPad Prism 5.0 (GraphPad Software Inc., La Jolla, CA, USA). For descriptive statistics, mean and standard deviation or median and interquartile range (IQR) were calculated. EORTC QLQ-C30 results were compared to established thresholds for clinical importance of the features to facilitate the interpretation of the results [23].

## Results

In this study n = 27 patients were initially screened for inclusion, of which n = 20 were included in the final analysis. Four patients were excluded due to lack of pelvic radiation, one patients due to diagnosis of fistula before radiation and two patients due to only conservative treatment (see Supplementary Fig. 1). At the time of the prospective data collection four patients had died, one patient suffered dementia, two patients could not be contacted and one patient did not want to take part in the data collection. Hence, 12 patients could be included in this part of the study.

The median duration of the radiation therapy in our cohort was 35 (34–41) days and the median radiation therapy dose was 75 (73–99) Gy. The median time from radiation therapy to diagnosis of pubic bone osteomyelitis and/or fistulas was 4.0 (0.9–6.5) years. The median time from first occurrence of symptoms until definitive surgery was 5.0 (1.7–15.5) months. Pubic bone osteomyelitis could be detected in 13 patients (65%) in the preoperative imaging and/or during surgery, fistulas could be observed in 19 patients (95%).

All patients underwent cystectomy or ureteral clipping, 10 patients (50%) underwent additional resection of the pubic symphysis or at least a surgical debridement of the pubic symphysis and eight patients (40%) needed an external fixation for stabilization of the pelvic ring. Temporary urinary diversion via urethral catheter or suprapubic tube before development of fistulas and/or pubic bone osteomyelitis was observed in 10 patients (50%). Preoperative urine cultures identified in 88% of the patients relevant levels of bacteria. Intraoperative microbiological smears of the pubic symphysis detected germs in 67% of the patients. Detailed demographic and clinical patient characteristics are summarized in Table 1

**Table 1:** Baseline demographics and clinical characteristics of the study population (n = 20)

Age in years, median (IQR)	73 (69–78)
Male, n (%)	19 (95)
ASA, median (IQR)	3 (2–3)
BMI kg/m^2^, median (IQR)	27.5 (25.3–31.0)
Basic oncological disease, n (%)	
Prostate cancer	17 (85)
Colorectal cancer	1 (5)
Anal cancer	1 (5)
Cervical cancer	1 (5)
Duration of radiation therapy (days), median (IQR)	35 (34–41)^a^
Cumulative dose of radiation (Gray), median (IQR)	75 (73–99)^a^
Type of radiation therapy, n (%)	
External beam radiation	19 (95)
Brachytherapy	1 (5)
Time from radiation to diagnosis (years), median (IQR)	4.0 (0.9–6.5)^b^
Time from first symptoms to diagnosis (months), median (IQR)	1.0 (1.0–1.0)
Time from first symptoms to surgery (months), median (IQR)	5.0 (1.7–15.5)^a^
Type of complication, n (%)	
Pubic osteomyelitis	13 (65)
Fistula	19 (95)
History of urinary catheter, n (%)	10 (50)
Preceding endoscopic treatment, n (%)	15 (75)
Transurethral incision	7 (35)
TURP	5 (25)
Bladder neck incision/resection	1 (5)
Artificial urinary sphincter implantation/explantation	3 (15)
Positive microbiological results, n (%)	
Urinary culture	15 (88)^a^
Smear of pubic symphysis	8 (67)^c^
Surgical treatment, n (%) Cystectomy/ureteral clipping	20 (100)
Pubic bone resection/debridement	10 (50)
External fixation	8 (40)
Urinary diversion, n (%)	
Ileal conduit	19 (95)
Ureteral clipping and percutaneous nephrostomy	1 (5)

*TURP* transurethral resection of the prostate

^a^Data of 3 patients missing

^b^Data of 1 patients missing

^c^Data of 8 patients missing

In most patients (85%) prostate cancer was the underlying oncological disease that led to pelvic radiotherapy at some point in the therapy sequence (see Supplementary Fig. 2).

**Fig. 1:**
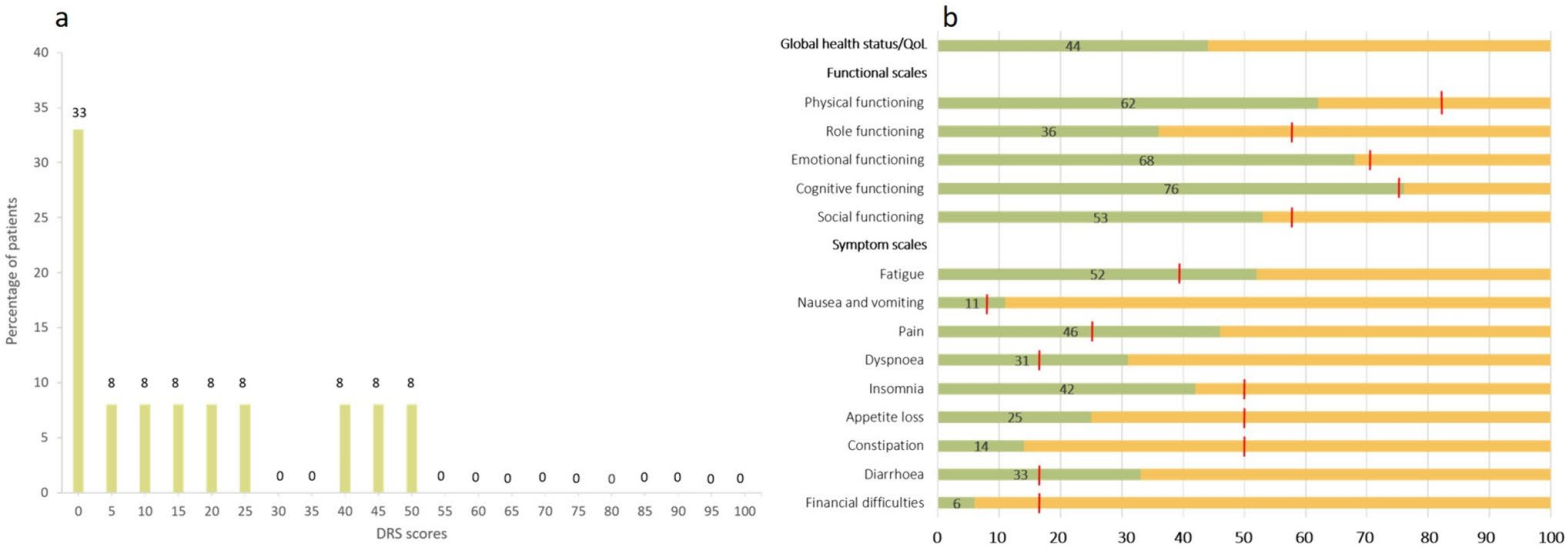
Results of the decision regret scale (**a**) and of the EORTC QLQ-C30 (**b**), red lines indicting thresholds for clinical importance; data of 8 patients missing

Patient-reported distress or remorse after the decision to undergo cystectomy revealed a mean DRS of 18 (± 19) points. The distribution of the scores is illustrated in Fig. 1a.

Evaluation of QoL, symptom and functional scales showed that nausea/vomiting, loss of appetite, insomnia and constipation were less pronounced after cystectomy and below the threshold for clinical significance, whereas physical and role functioning and symptoms such as fatigue, pain, dyspnoea and diarrhoea seemed to be more affected after cystectomy, as they occurred more frequently compared to the established thresholds for clinical importance. Overall QoL/global health status, functional and symptom scales of the EORTC QLQ-C30 are shown in Fig. 1b. The thresholds for clinical importance of the functional and symptom scales are marked in the figure [23]. The questionnaires were completed on average 23 months after cystectomy.

**Fig. 2:**
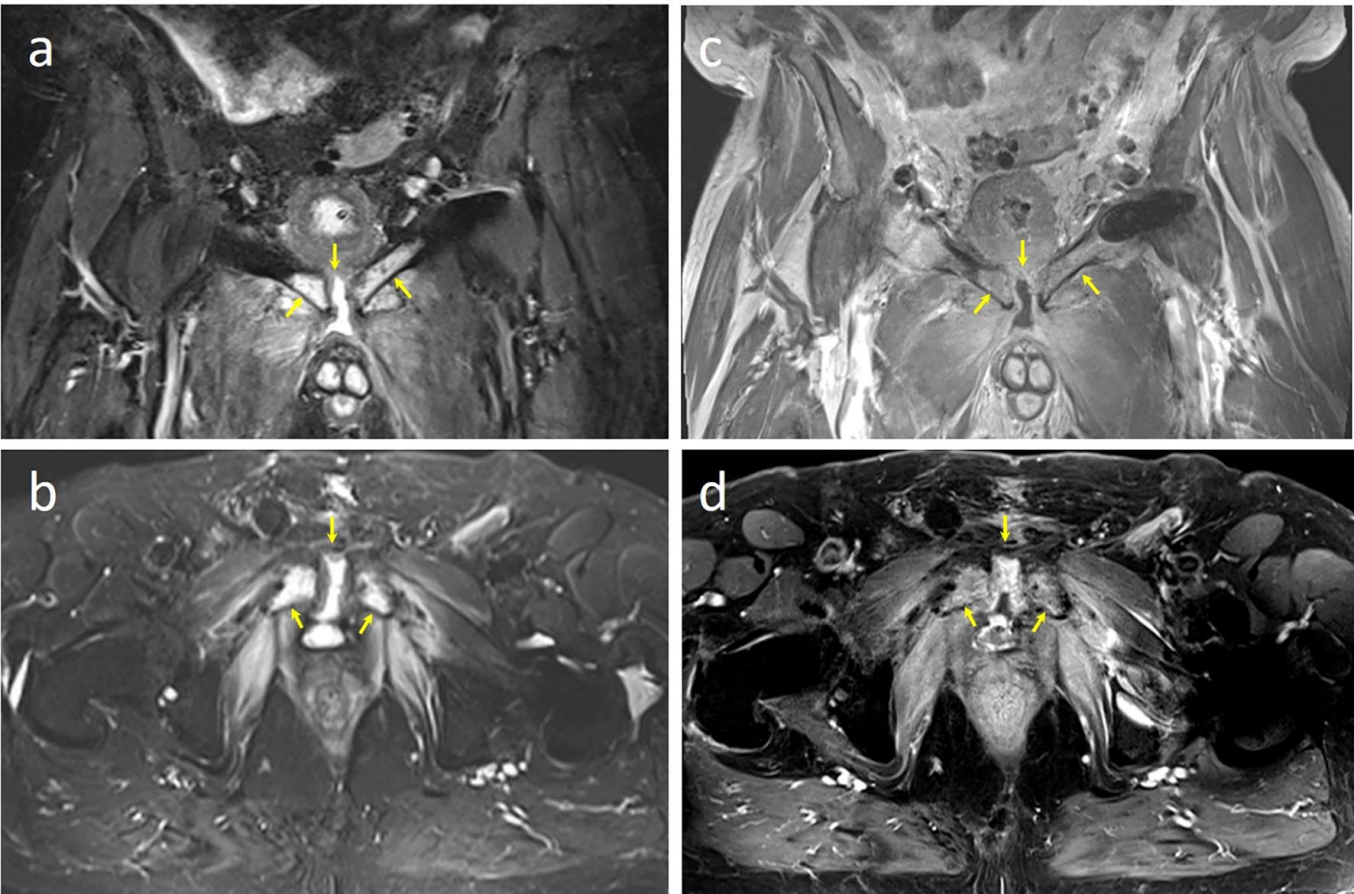
76-year old male patient with acute pubic bone osteomyelitis. **a** Coronal T2/STIR and **b** axial T2 TSE fs images depict marked bilateral edema of the pubic bones and widening of the symphyseal cleft with fluid collection. **c** Coronal T1 non-fat sat and **d** axial T1 fat-sat post-contrast images yield diffuse bilateral contrast enhancement of the pubic bone, of the adductor muscle group and of the internal obturator muscle. These findings are in keeping with pronounced acute pubic bone osteomyelitis, the patient received an overall score of 5 in the radiological read and subsequent symphyseal resection

The results of the radiological image analysis showed a mean point score of 1.1 points in the imaging after radiotherapy and before onset of symptoms, 2.2 points after first occurrence of symptoms and 3.3 points in the imaging before reconstructive surgery. Both radiologists assigned the same point score for the imaging after radiotherapy and before first occurrence of symptoms and after beginning of first symptoms. Only in the imaging before definitive surgery the assigned points differed slightly (3.2 vs.3.5 points, see Supplementary Fig. 3). Figure 2 illustrates MR imaging of a patient with pronounced signs of acute pubic bone osteomyelitis.

## Discussion

Due to an aging society and improved cancer therapy, more patients undergo pelvic radiation for tumors during long-term survivorship. Consequently, there's a rising incidence of late UAEs post-pelvic radiation [8]. Pelvic osteomyelitis and urosymphyseal fistulas in this context are rare, with poorly documented incidence and prevalence. Most pre-2010 studies were limited case series. According to German health insurance data, 5.7% of patients receiving pelvic radiation for tumors between 2010 and 2017 developed late UAEs.

Patients with these complications often undergo prolonged multimodal treatments, where conservative approaches prove ineffective. The transition to radical therapy through cystectomy and urinary diversion is delayed, reflecting years of suffering. In our cohort, 95% opted for cystectomy with ileal conduit, while 5% chose bladder-sparing surgery. Additional pubic bone resection was performed in 50% and 40% required debridement with external fixation, aligning with prior studies favoring cystectomy for post-pelvic radiation fistulas and pubic bone osteomyelitis [24].

Following a median radiation duration of 35 (34–41) days, mirroring the standard external beam radiation therapy for prostate cancer, our cohort showed time frames of 4.0 (0.9–6.5) years from radiotherapy completion to diagnosis, 1.0 (1.0–1.0) months from symptom onset to diagnosis and 5.0 (1.7–15.5) months from symptom onset to definitive surgical therapy. This latency, ranging from years to over a decade, is consistent with findings from Toia et al., reporting a median 6-year interval to primary urological reconstructive surgery post-pelvic radiation [12, 25]. Faris et al. observed a median 4.6-year duration from radiation therapy to symptom presentation in prostate cancer patients [26]. Our median cumulative pelvic radiation dose 75 (73–99) Gy aligns with standard therapy for prostate cancer, as seen in studies reporting comparable doses [27]. However, variations in doses, treatment schedules, and follow-up durations across studies limit direct comparisons regarding the toxic effects of the applied radiation dose.

Temporary urinary diversion and a history of endoscopic treatments are recognized risk factors for complications post-pelvic radiation. In our cohort, 75% underwent urethral manipulations, aligning with Andrews et al.’s findings [28]. Cystectomy is often the preferred medical choice, with patient-reported outcomes revealing no regrets and 36% of the patients of our cohort having no doubts at all that cystectomy was the right decision. No patient showed a score of more than 50 in the DRS which is underlining the tendency towards the patients’ well-being after cystectomy. Functional outcomes vary, with cognitive and emotional functioning showing improvement, while physical and role functioning may be more affected. Symptoms post-cystectomy exceed clinical thresholds in fatigue, nausea, pain, dyspnea, and diarrhea, while appetite loss, insomnia, and constipation are less pronounced. These findings partly align with studies reporting enhanced postoperative functional status, reduced pelvic pain, and improved pain scores [10, 16, 24, 25, 28]. Andrews et al. demonstrated significant postoperative functional status restoration [28]. Our cohort's assessment, with a median time of 25 months between cystectomy and EORTC QLQ-C30 evaluation, may reflect additional comorbidities. Cognitive and emotional outcomes align with stable mental health post-cystectomy in prostate cancer survivors [25].

Our radiological assessment demonstrated a progressive increase in the score over time. Pre-symptomatic CT and MRI scans exhibited minimal tissue and bone alterations. Upon symptom onset, imaging revealed tissue changes consistent with pubic bone inflammation, resulting in an elevated score. Imaging immediately before surgery depicted the highest score, signifying a progression in specific alterations. Radiographically, MRI emerged as the most precise technique for detecting changes consistent with pubic bone osteomyelitis and urosymphyseal fistulas [19, 20, 29, 30]. While CT scans reveal osseous changes and signs of chronic osteomyelitis, they are less sensitive than MRI due to decreased soft tissue contrast [19]. Nevertheless, CT contributes valuably to rapid diagnosis, given its detectable osteomyelitis characteristics and faster availability compared to MRI. Our data underscore the notion that early imaging, particularly MRI, serves as a reliable diagnostic tool for suspected pelvic osteomyelitis, facilitating optimal surgical planning. Implementing a point score for CT and MR scans could assist in classifying pubic bone osteomyelitis severity, expediting definitive treatment. Radiologists engaged in such cases should be well-acquainted with the imaging features of late UAEs and anticipated post-treatment findings.

Our study is not exempt from limitations, with the primary constraint being the modest sample size, which can compromise the representativeness and generalizability of our findings. The absence of a control group poses another limitation, and its inclusion would augment internal validity by mitigating the impact of confounding variables. Additionally, limited blinding during radiological assessment introduces potential bias in CT and MRI scan scoring. Furthermore, our reliance on data from a solitary German health insurance company, with a three-year follow-up, may underestimate late UAE occurrences beyond this period. Despite these limitations, our study contributes valuable insights into the trajectory, diagnosis, and management of late UAEs post-pelvic radiation.

## Conclusion

The diagnosis of pubic bone osteomyelitis and urosymphyseal fistulas after radiotherapy of the pelvic region requires a high index of suspicion and an accurate and thorough evaluation. Conducting an extensive diagnostic workup, including early imaging followed by accurate assessment of the typical characteristics of pubic bone osteomyelitis, reduces the time frame to diagnosis and to definitive treatment. Thereby, long suffering could be prevented and QoL preserved. Interdisciplinary follow-up care, including urological assessment, after pelvic radiation of also non-urological tumors, should be standard and multidisciplinary therapy approaches are crucial for a successful management of these complications.

## Supplementary Information

Below is the link to the electronic supplementary material.

Supplementary file1 Supplementary Fig. 1 Flow chart of exclusion criteria and cohort size (PDF 210 KB)

Supplementary file2 Supplementary Fig. 2 Therapy sequences of the cohort; RPX = radical prostatectomy, XRT = (pelvic) radiation therapy, HIFU = high-intensity focused ultrasound, LAR = low anterior resection, LAE = pelvic lymphadenectomy; *anal cancer as primary disease, #colorectal cancer as primary disease, +cervical cancer as primary disease (PDF 201 KB)

Supplementary file3 Supplementary Fig. 3 Results of the radiological assessment of CT and MRI scans of the study cohort performed by both radiologists (a+b) (PDF 206 KB)

## Data Availability

All data generated or analyzed during this study are included in this article. Further enquiries can be directed to the corresponding author.

## Abbreviations

ASA: American Society of Anesthesiologists
BMI: Body mass index
CI: Confidence interval
CT: Computed tomography
Gy: Gray
IQR: Interquartile range
MRI: Magnet resonance imaging
QoL: Quality of life
SD: Standard deviation
UAE: Urinary adverse event
