# Supplementary figures and images for: Pubic bone osteomyelitis and fistulas after radiation therapy of the pelvic region: patient-reported outcomes and urological management of a rare but serious complication
Source: World J Urol. 2024 Aug 1;42(1):461. doi: 10.1007/s00345-024-05155-2 (PMC11294262; doi:10.1007/s00345-024-05155-2)

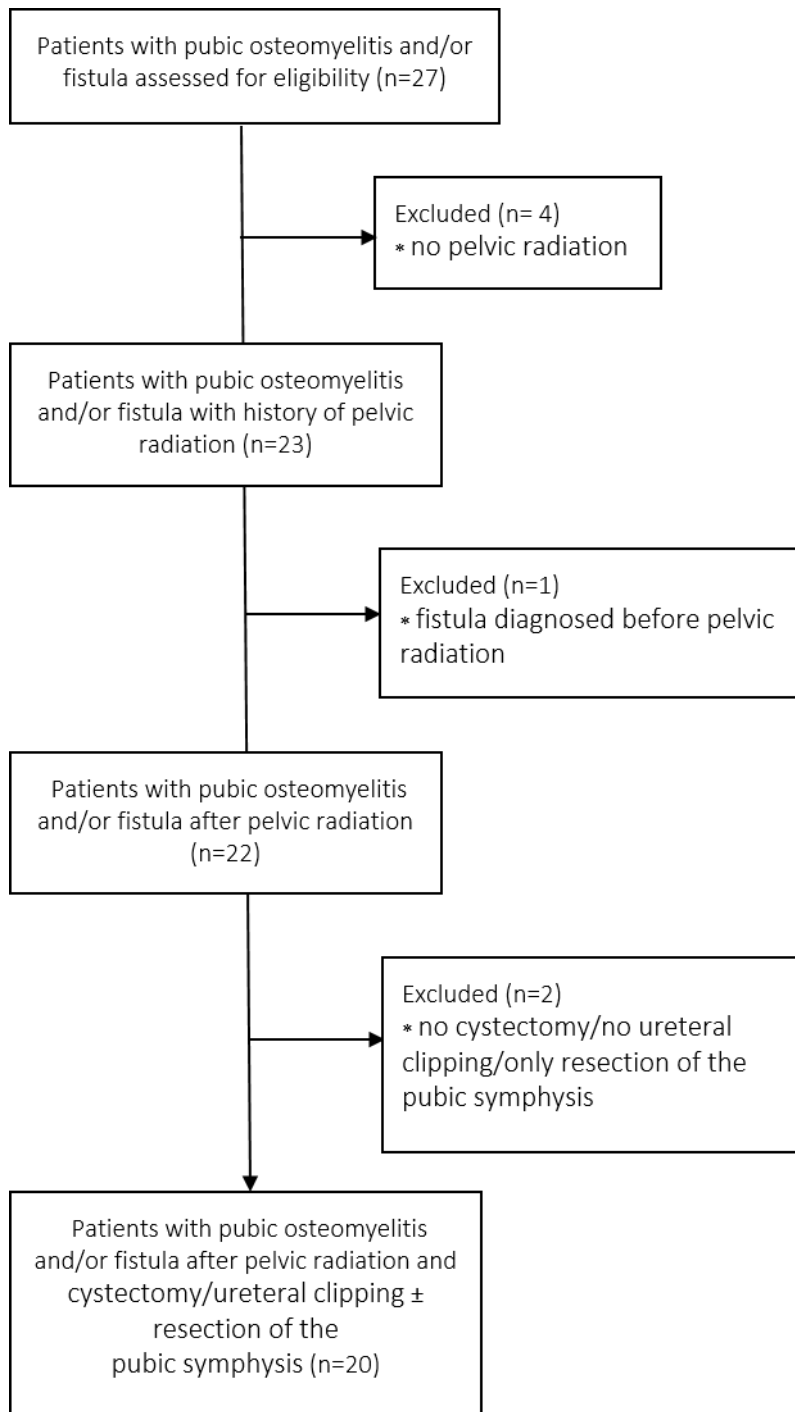

Supplement: Supplementary file 1 — Supplementary file1 Supplementary Fig. 1 Flow chart of exclusion criteria and cohort size (PDF 210 KB) [file 345_2024_5155_MOESM1_ESM.pdf]

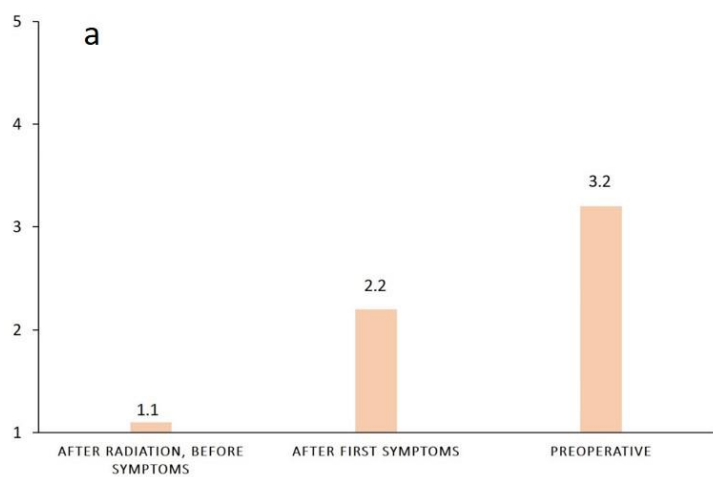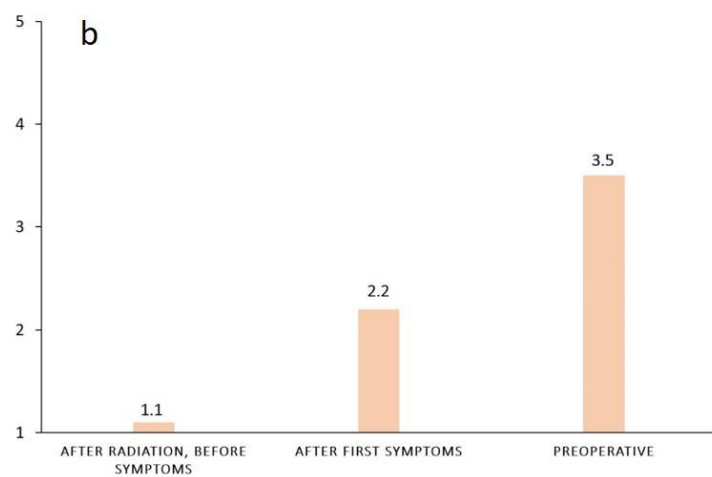

Supplement: Supplementary file 3 — Supplementary file3 Supplementary Fig. 3 Results of the radiological assessment of CT and MRI scans of the study cohort performed by both radiologists (a+b) (PDF 206 KB) [file 345_2024_5155_MOESM3_ESM.pdf]
