# Supplementary material for: Pubic bone osteomyelitis and fistulas after radiation therapy of the pelvic region: patient-reported outcomes and urological management of a rare but serious complication
Source: World J Urol. 2024 Aug 1;42(1):461. doi: 10.1007/s00345-024-05155-2 (PMC11294262; doi:10.1007/s00345-024-05155-2)
Supplement: Supplementary file 2 — Supplementary file2 Supplementary Fig. 2 Therapy sequences of the cohort; RPX = radical prostatectomy, XRT = (pelvic) radiation therapy, HIFU = high-intensity focused ultrasound, LAR = low anterior resection, LAE = pelvic lymphadenectomy; *anal cancer as primary disease, #colorectal cancer as primary disease, +cervical cancer as primary disease (PDF 201 KB) [file 345_2024_5155_MOESM2_ESM.pdf]

n = 13	RPX	→ XRT	→	Pubic bone osteomyleitis/fistula
n = 1	HIFU	→ XRT	→	Pubic bone osteomyleitis/fistula
n = 1	XRT	→ RPX	→	Pubic bone osteomyleitis/fistula
n = 2	XRT	→	→	Pubic bone osteomyleitis/fistula
n = 1*	LAR	→ XRT	→	Pubic bone osteomyleitis/fistula
n = 1[#]	XRT	→ LAR	→	Pubic bone osteomyleitis/fistula
n = 1[+]	LAE	→ XRT	→	Pubic bone osteomyleitis/fistula
